# Combining Fluorescent Cell Sorting and Single B Cell Amplification to Screen the Monoclonal Antibody Gene against Human Glypican-1 in Pancreatic Cancer

**DOI:** 10.1155/2021/5646589

**Published:** 2021-09-03

**Authors:** Mi Huang, Yingying Ma, Xiaoyan Gao, Xinyang Li, Quan Ding, Chuxin Liu, Xiaopan Liu, Hang Zhang, Naibo Yang

**Affiliations:** ^1^BGI-Shenzhen, Shenzhen, Guangdong 518083, China; ^2^BGI-Wuhan, Wuhan 430074, China; ^3^BGI Education Center, University of Chinese Academy of Sciences, Shenzhen, Guangdong 518083, China; ^4^Complete Genomics Inc., San Jose, CA, USA

## Abstract

In this report, one novel method has been developed to screen the monoclonal antibody against human pancreatic cancer biomarker glypican-1 (GPC1) through the combination of fluorescent cell sorting and single B cell amplification. GPC1-positive B cells were sorted out from the peripheral blood mononuclear cells (PBMCs) by fluorescent cell sorting after the GPC1 immunization to the New Zealand white rabbit. Then, total RNA was extracted and reversely transcribed into cDNA, which was used as the template, and the variable region sequences of both heavy and light chains were amplified from the same B cell. Next, their recombinant antibody was expressed and purified from the human 293T cell after the antibody gene amplification and expression vector construction. The enzyme-linked immunosorbent assay (ELISA) and flow cytometry assays were used to determine the antibody affinity. The antibody named GPC-12 that we screened and obtained was proven to have natural heavy-light chain pairing information, and it was highly specific to the GPC1 antigen, and the affinity could reach 1 × 10^−7^ M. Overall, an effective and novel method has been successfully developed to screen the antibody by combining the fluorescent cell sorting and single-cell amplifying technologies, which was proved to be workable in our setting.

## 1. Introduction

Pancreatic cancer is a malignant gastrointestinal malignancy, with the prognosis being rather grim [1]. In recent years, the incidence of pancreatic cancer has been shown to have an upward trend, and it is predicted that pancreatic cancer causes more than 331,000 deaths every year [2, 3]. Overall survival rate of pancreatic cancer ranked the lowest among all kinds of malignant tumors. The five-year survival rate was only 2%–9%, with an average being no more than 6% [2], and the morbidity/mortality rate was close to 100% [4]. Therefore, it is essential to develop the new diagnostic method for pancreatic cancer, known as the king of cancer, which makes the early diagnosis of pancreatic cancer become possible.

The diagnosis of pancreatic cancer is mainly based on a comprehensive diagnostic method, that is, imaging, tumor markers, and tissue biopsy [5]. However, the diameter of early pancreatic cancer is usually less than two centimeters; and it is confined to the pancreatic parenchyma and does not invade the pancreatic capsule. Traditional imaging methods are not sensitive and specific enough to detect pancreatic cancer lesions. All these make it extremely difficult to measure and detect the pancreatic cancer. Therefore, it is required to develop the novel method to earlier detect the multiple tumor markers, which plays an important role in improving the positive detection rate of cancer patients, cancer screening in high-risk groups without clinical symptoms, and early detection of pancreatic cancer [6, 7].

At present, CA19-9 and CEA are commonly used tumor markers for detection of pancreatic cancer, but the two biomarkers were less specific in that they are also related antigens in other cancers, such as colorectal cancer or ovarian cancer [8, 9]. The positive rate of CA19-9 was over 80%, but the positive rate of CA19-9 in small pancreatic cancer patients was not high (60.7% in adenocarcinoma patients less than 2 cm), while the positive rate of CEA was only 78.9%. Therefore, there is an urgent need to find a more specific antibody for pancreatic cancer, that is to say, a detection reagent with higher diagnostic positive rate, which can be used as a common detection marker for pancreatic cancer [10, 11]. As early as 1998, it has been reported that Glypican-1 (GPC1) was overexpressed in pancreatic cancer cells; and that Glypican-1 could regulate the metastasis and angiogenesis of pancreatic cancer cells, playing a pivotal role in the oncogenesis of pancreatic ductal adenocarcinoma [12]. These previous findings suggest that GPC1 can be a potential diagnostic biomarker. In addition, in 2015, one study reported that GPC1 had nearly 100% sensitivity and specificity in both animal models and clinical patients with pancreatic cancer, being able to accurately detect early pancreatic cancer, indicating that GPC1 has great potential as a tumor marker for early detection of pancreatic cancer [13–15].

Consequently, to screen the monoclonal antibody against human pancreatic cancer biomarker GPC1, in this study, a single B cell clone against human pancreatic cancer GPC1 was sorted out by flow cytometry; and then a natural pair of antibody heavy and light chain sequences were amplified from a single B cell by PCR. In performing PCR, the codons preferred by adult mammalian cells were optimized. After gene synthesis, the expression vector was constructed and transfected into HEK293T cells for expression. The binding activity of antibody to antigen was evaluated by ELISA, and the affinity kinetics between antibody protein and antigen GPC1 were verified by surface plasmon resonance (SPR).

## 2. Materials and Methods

### 2.1. Materials

New Zealand white rabbits were purchased and reared in the Food and Drug Safety Evaluation Center of Hubei Province. Sample homogenate device (instrumental model: T10) was purchased from IKA-Werke GmbH & Co. KG (Staufen, Germany). Analytical flow cytometry (CytoFLEX S) was purchased from Beckman Coulter Inc. (Beckman Coulter, Brea, CA, USA). Sorting flow cytometry (MoFlo XDP) was purchased from Beckman Coulter Inc. (Beckman Coulter, Brea, CA, USA). Horizontal hanging basket centrifuge (H1850R) was from Hunan Xiangyi, and 1 × RPMI1640 basic medium was purchased from Gibco Company. Fetal bovine serum (FBS) was from HyClone Company. Horseradish peroxidase labeled sheep anti-rabbit IgG was purchased from GeneTex Company. SuperScript II Reverse Transcriptase was purchased from Invitrogen Inc. (Life Technologies, Carlsbad, CA, USA). Fluorescein Isothiocyanate (FITC) was purchased from Sigma Chemical Co. (St. Louis, MO, USA). Ficoll-Paque PREMIUM for PBMC separation was purchased from GE Healthcare (San Diego, CA, USA). D2000 DNA ladder was purchased from Tiangen Biochemical Technology Co., Ltd. (Beijing, China). rTaq enzyme and TA clone are from TaKaRa (TaKaRa, Japan) with pMD18-T vector, *E. coli* competent cell DH5-alpha, and template switch oligos (TSO). Master Mix was purchased from Kapa Biosystems (Boston, MA, USA). Bovine serum albumin, Triton X-100, Freund's complete adjuvant, Freund's incomplete adjuvant, and GPC1 polypeptide were purchased from Sigma Chemical Co. (St. Louis, MO, USA). PCR product purification kit and agarose gel recovery kit were purchased from QIAGEN Company (Germany). pFUSE-Fc mammalian cell expression plasmid was purchased from Invitrogen Inc. (Life, Carlsbad, CA, USA). Commercial anti-GPC1 monoclonal antibodies were purchased from GeneTex (CA, USA).

### 2.2. Animal Immunization

The experimental animal immunization and blood collection program were approved by the Animal Ethics Committee of Shenzhen Huada Academy of Life Sciences (ethics approval number FT15149). Two New Zealand white rabbits aged 5-6 weeks were immunized by subcutaneous injection on the back. They were immunized once every two weeks, four times in total. 500 *μ*l antigen (200 micrograms of antigen protein dissolved in 500 *μ*l of sterile water to form a solution of 0.4 mg/mL) and 500 *μ*l Freund's complete adjuvant were used uniformly. The pulp machine emulsified evenly and was used for the first time before immunization. The other three immunizations used a mixture of Freund's incomplete adjuvant and antigen emulsifies evenly and equally.

### 2.3. Blood Collection and Cell Separation

Before or two weeks after the final immunization, blood samples were collected from the ear vein of two white rabbits. The serum of 100 *μ*l before and after immunization was used to test the titer of anti-positive antibody. The peripheral blood of a white rabbit with strong immune response was collected and separated by Ficoll lymphocyte gradient centrifugation.

### 2.4. Detection of Serum and Antibody Titers by ELISA

The antigen was dissolved in coating solution (50 mM Na_2_CO_3_, pH 9.6) at an appropriate concentration, and 100 *μ*l antigen was added to the corresponding pore overnight at 4°C. The liquid was discarded and the residual liquid was dried, followed by rinsing with the detergent PBST (1 × PBS pH 7.4 plus 0.05% Tween 20, the same as below) three times. Each pore was incubated at 37°C for 1 h with 200 *μ*l blocking solution (3% BSA). The liquid was emptied and the remaining liquid was dried. The washing liquid was used for rinsing three times. Each pore was incubated with 100 *μ*l diluted serum or antibody, at 37°C for 1 hour. The liquid was emptied and the remaining liquid was dried. The washing liquid was used for rinsing three times. Each pore was incubated with 100 *μ*l secondary antibody (1 : 5000 diluted horseradish peroxidase labeled sheep anti-rabbit IgG) for 1 hour at 37°C. The liquid was emptied and the remaining liquid was dried. The washing liquid was used for rinsing five times. The residual liquids in the dry holes were patted, and 100 *μ*l color developing solution was added to each hole, and the color was visualized for 10 minutes without light. Each hole was terminated by adding 50 *μ*l 2M H_2_SO_4_, and the 450 nm OD value was read immediately.

### 2.5. Cell Staining and Selection

The 1 mg GPC1 antigen was dissolved in 0.1 M carbonate buffer of 0.5 mL pH9.0. FITC (sigma F4274) was dissolved with DMSO, and the final concentration of FITC was 1 mg/mL. FITC-DMSO solution was slowly added to antigen solution, and 50 *μ*l FITC-DMSO solution was added to antigen solution. The reaction time was 8 h at 4°C. The reaction was terminated by adding NH_4_CL with the final concentration of 50 mM and reacted at 4°C for 2 h. Ultrafiltration removes unreacted FITC.

The isolated rabbit PBMC was suspended to the final volume of 20 *μ*l PBS and 2 *μ*l GPC1-FITC was added. The PBMC was gently vortexed and blended. The light-avoiding reaction lasted 1 hour at room temperature (a small number of cells without GPC1-FITC were reserved as negative control). After the reaction, 1 mL PBS was added; the supernatant was discarded by centrifugation for 5 minutes and precipitated by 1 mL PBS. The supernatant was discarded by centrifugation for 5 minutes and precipitated by 1 mL PBS. The samples were prepared by centrifugation for 5 min at 400 ×g, discarding the supernatant, and resuspension precipitation with 500 *μ*l PBS. The density of the samples was about 10^7^/mL.

### 2.6. Preparation of Cell Lysate

A 4 *μ*l lysate [1.86 *μ*l nonnuclease water, 1 *μ*l Oligo(dT)18 (10 mu-ol/L), 0.1 *μ*l RNase inhibitor (4 U), and 0.04 *μ*l Triton X-100 (100 mL/L)] was prepared. The lysate was placed in a 0.2 mL PCR tube (8 rows of 96-well plate). The selected cells were directly added to the cell lysate. One tube of negative pore (0 cells, i.e., no cell sorting) was reserved for each row; one tube of positive pore (i.e., 10 cells sorted) was used as control, and 10 tubes of single-cell pore were reserved for each row. Twelve PCR tubes can be selected as one group for each experiment, and the rest can be frozen to −80°C group. After two weeks of use, the success rate of amplification will be greatly reduced.

### 2.7. Separation of GPC1-FITC-Positive Cells by Flow Cytometry

Conventional flow cytometry was subjected to calibration, and then calibration was undertaken for sorting parameters; the latency value of side fluid flow and droplets were adjusted to make the left bright spot farthest and most concentrated in the side fluid flow window; then the coordinate value of cell sorting around the coordinate window was adjusted to ensure that the sheath fluid can hit the bottom of the PCR tube (cells cannot hit the wall of the side tube; otherwise, it would affect the success rate of amplification). At last, the rate of cell sorting was checked, such as setting 10 cells to be sorted; looking at the actual number of cells under the microscope, more than 90% of the cells were qualified and could be sorted in the next step. After all the above steps were completed, the sorting commenced.

### 2.8. Cell Lysis and Reverse Transcription

The PCR tube containing lysate was placed in the PCR instrument at 72°C for 3 minutes; the hot cover was set at 75°C for 1 min on ice immediately after lysis and centrifuged at 10,000 r/min for 30 seconds. 6 *μ*l reverse transcription system [2 *μ*l SuperScript II First-Strand Buffer (5×), 2 *μ*l betaine (5 mol/L), 0.9 mu-L MgCl_2_ (100 mmol/L), 0.25 *μ*l DTT (100 mmol/L), 0.1 *μ*l TSO (100 *μ*mol/L), 0.25 *μ*l RNA inhibitor (4 U), and 0.5 *μ*l SuperScript II Reverse Transcriptase (20 U)] was prepared. Reverse transcription was performed under the following conditions: 42°C for 90 min, 50°C for 2 min, 42°C for 2 min, 10 cycles, 70°C for 15 min, and 12°C. The DNA was obtained.

### 2.9. Amplification of the Antibody Sequence

Primer Premier 5 software was used to design primers. The products of single-tube cDNA system obtained by the above methods were used as templates for two rounds of nested PCR. In the first round, Ld primers (rIgH-Ld1, rIgK-Ld1, rIgL-Ld1-1, and rIgL-Ld1-2) and downstream primers of heavy and light chain C region (rIgHG-C1, rIgK-C1, and rIgL-C1) were used to prepare 25 *μ*l PCR system [12.5 *μ*l KAPA ReadyMix (2×∗), 0.5 *μ*l Ld primers (10 *μ*mol/L), 10 *μ*l reverse transcription products, and 1.5 *μ*l nuclease-free water]. The second round of PCR used FR primers (rIgH-FR1 and rIgK-FR1) and J primers (rIgH-J, rIgK-J-1, rIgK-J-2, and rIgL-J). A 25 *μ*l PCR system [12.5 *μ*l KAPA ReadyMix (2×), 0.5 *μ*l FR primer (10 *μ*mol/L), 0.5 *μ*l J primer (10 *μ*mol/L), 1 *μ*l first-round PCR product, and 10.5 *μ*l ribonuclease-free water] was prepared. The amplification conditions for both rounds of PCR were as follows: 95°C for 3 min, 98°C for 15 s, 60°C for 20 s, 72°C for 2 min, 25 cycles, 72°C for 5 min, and 12°C. The amplification system could be amplified as needed. Here, a nested amplification primer was designed (Table 1), TSO and oligo(dT) primers were used in the reverse transcription stage; Ld and C primers were annealed to the upstream sequence of TSO and the downstream CH1 region of antibody subtypes in the first round of PCR, respectively, and the primers of 10 mm/L were mixed equally; the other primers were the second round of PCR primers.

### 2.10. TA Cloning, Gene Synthesis, and Expression Vector Construction

10 g/L agarose gel was used to detect the amplified products of PCR, and the target bands (380 BP) were cut and recovered by gel recovery kit. Then, rTaq enzyme was used to add A to the end of the gel-cut recovery product, and the product was purified by the PCR product purification kit and then linked to the pMD18 T vector overnight at 16°C. The conjugates were transferred into competent cells DH5*α* and cultured in antibiotic-free Luria-Bertani (LB) medium at 37°C for 1 hour. The conjugates were coated on ampicillin-containing LB solid medium overnight. The next day, the monoclonal colony in good condition was selected for shaking culture for 3 to 5 hours, and then the liquid was sent to Shanghai Shanggong Company for DNA sequencing to verify whether the antibody sequence is correct and also to verify whether the expression contains both heavy chain and light chain in the correct pairing. According to the preference of human cell expression system, the codon of the amplified heavy and light chain genes was optimized, and then the whole gene of variable region was synthesized and cloned directly into the commercial mammalian cell expression vector pFUSE-rabbit Fc, which contained the Fc constant region of rabbit antibody.

### 2.11. Expression of Mammalian Cells and Purification of the Antibody

Trypsinase digestive cells were counted and paved and cultured overnight at 37°C and 5% CO_2_, and the confluence of cells was 70%–80% at transfection. The plasmid was diluted with a certain volume of Opti-MEM (Invitrogen 11058-021), carefully mixed, and labeled as liquid A. The transfection reagent PEI (Polyscience 23966-2) was diluted with the same volume of Opti-MEM, named liquid B. The mass ratio of plasmid to PEI was 1 : 2. It was kept at room temperature for 15 minutes. Liquid B was slowly added to liquid A and it was mixed. It was kept at room temperature for 20 minutes. The DNA-PEI mixture was slowly added to the cell culture medium and it was gently mixed. 48 hours after transfection, the supernatant was collected and centrifuged for 5 minutes to remove the cells. The supernatant was centrifuged for 10 minutes to remove the cell debris. The antibody was purified by affinity purification column coupled with GPC1 antigen.

### 2.12. Antigen-Antibody Binding Kinetics (Affinity Test)

The GPC1 antigen was dissolved in the coating solution (50 mM Na_2_CO_3_, pH 9.6) at an appropriate concentration, and 100 *μ*l antigen was added to the corresponding pore for overnight stay at 4°C. The liquid was emptied and the residual liquid was pat-dried; the PBST was washed (PBST and pat dry the residual liquid, add 1 mL/L Tween 20, the same as below) and rinsing was performed three times. Each pore was incubated with 200 *μ*l blocking solution (3 g/L bovine serum albumin) for 2 hours at 37°C. The liquid was emptied and the remaining liquid was dried. Wash the PBST detergent was washed three times. Each pore was incubated at 37°C for 1 hour with 100 *μ*l diluted antibody. The liquid was emptied and the remaining liquid was dried. The washing liquid was used for rinsing three times. Each pore was incubated with 100 *μ*l secondary antibody (1 : 5000 diluted horseradish peroxidase labeled sheep anti-rabbit IgG) for 1 hour at 37°C. The liquid was emptied and the remaining liquid was dried. The washing liquid was used for rinsing five times. The residual liquid in the dry hole was patted, adding 100 *μ*l color developing solution to each hole, and coloring at 37°C for 5 minutes. Each hole was added with 50 *μ*l 2M HCL to stop the color rendering and 450 nm OD value was read immediately.

### 2.13. Detection of Antibody Binding to Natural GPC1 on the Cell Surface

MDA-MB-231 (China Typical Culture Preservation Center) in logarithmic growth phase was digested into single-cell suspension, counted, and centrifuged to remove the medium. Cell suspension was separated into three centrifugal tubes according to 50 *μ*l/tube with proper volume of PBS suspension cells. No. 1 was without antibody, No. 2 was added with 0.5 *μ*l positive GPC1 antibody (GeneTex GTX42664), and No. 3 was added with 1 *μ*l purified GPC1 antibody and incubated at room temperature for 30 minutes. Each tube was centrifuged with 1 mL PBS for 5 min at 300 ×*g* for supernatant. 1 mL PBS suspended cells were precipitated and centrifuged for 5 min at 300 ×*g*, and then the supernatant was discarded twice. The 50 *μ*l PBS suspended cells were precipitated and incubated at room temperature for 30 minutes with the corresponding fluorescent labeled antibody. Each tube was centrifuged with 1 mL PBS for 5 min at 300 ×*g* for supernatant for three times. Flow detection was done on computer.

### 2.14. Statistical Analysis

All data were analyzed using SPSS 21.0 statistical software. The data were expressed as mean ± standard deviation. One-way ANOVA (Bonferroni) was used for comparisons among multiple groups. Tukey's post hoc test was used for pairwise comparisons of the mean values between multiple groups. Independent-sample *t*-test was used to analyze the difference between two groups where the data had normal distribution. *P* < 0.05 was considered to be statistically significant.

## 3. Results

### 3.1. Detection of the Serum-Positive Conversion Titer

To make the titer of the antibodies we produced clear, ELSIA was performed. As shown in Table 2, the blank group refers to the blank control without serum and with 100 *μ*l PBS. The negative group was the negative control with 100 *μ*l serum before immunization. The immune group refers to the serum diluted by 100 *μ*l ratio after the fourth immunization. The positive detection group still had a high absorbance value from 1 : 1000 dilution to 1 : 32000, and the positive/negative ratio was >8 (generally, the value was greater than 4; that is to say, the immune serum was positive), which indicated that specific antibodies against human GPC1 had been produced in rabbits, and Rabbit 1 was selected for the next experiment.

### 3.2. Cell Staining and Flow Cytometry

**Figure 1:**
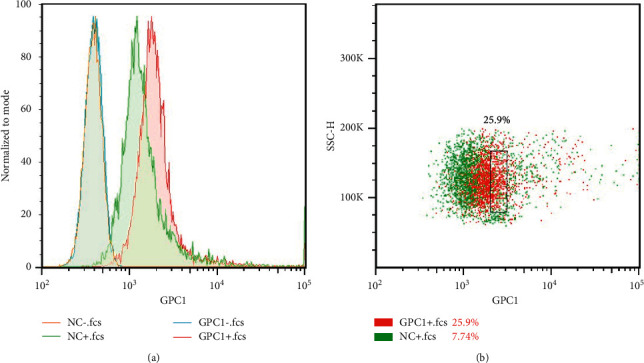
Cell fluorescent staining and sorting of single cell by flow cytometry. (a) Cell fluorescent staining; NC− means lymphocytes without GPC1-FITC staining. The lymphocytes were sampled from rabbits without immunization. NC+ means lymphocytes stained with GPC1-FITC. The lymphocytes were sampled from rabbits without immunization. GPC1− represents lymphocytes that were not stained with GPC1-FITC from immunized rabbits; GPC1+ denotes lymphocytes that were stained with GPC1-FITC from immunized rabbits. (b) B cell sorting by flow cytometry. GPC1+ cells were all that we want which were sorted by flow cytometry.

To stain fluorescently the cells of interest, GPC1-FITC was used. As shown in Figure 1(a), NC- was not stained with GPC1-FITC for immunized rabbit lymphocytes, NC+ was stained with GPC1-FITC for immunized rabbit lymphocytes, GPC1 was not stained with GPC1-FITC for immunized rabbit lymphocytes, and GPC1+ was stained with GPC1-FITC for immunized rabbit lymphocytes. It can be seen that the lymphocyte without GPC1-FITC staining was in the same fluorescence intensity range regardless of immunization, and the lymphocyte without GPC1-FITC staining also showed positive deviation and nonspecific binding, but the fluorescence intensity was lower than that of immunized rabbit lymphocyte. Therefore, this part was the cells that need to be further sorted, as shown in Figure 1(b).

### 3.3. Amplification and Sequencing of the Monocyte Antibody Gene

**Figure 2:**
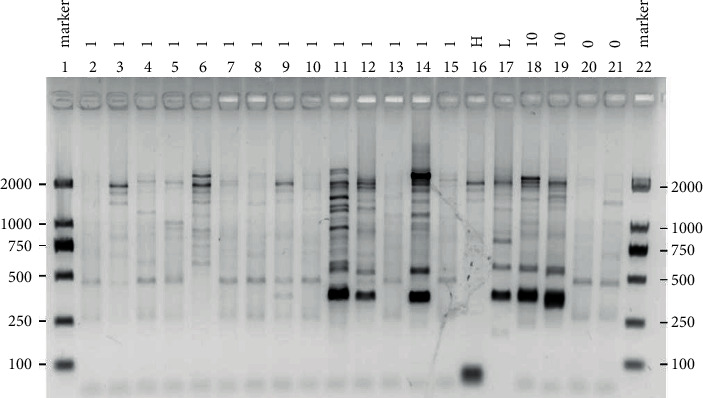
Electrophoresis of single-cell amplification. As shown in the electrophoretic gel, lanes 2–15 denote that only one cell sorted was used as the PCR template; lanes 18 and 19 were set as the positive control, where 10 cells sorted were utilized as the PCR template; lanes 20 and 21 were set as the negative control in which no cell was used in PCR. The gene of interest was around 380 bp. Lanes 11, 12, and 14, as well as positive controls, in which the bands of interest can be visibly achieved can be seen.

To purify the cells that were fluorescently stained with GPC1-FITC, cell sorting was employed based on flow cytometry. As shown in Figure 2, one cell was selected from lane 2 to lane 15 as a PCR template, from 18 to 19 as a positive control, 10 cells were selected as templates, and 20 to 21 as a negative control, which was no cells. The size of target protein was about 380 bp. It can be seen that, in lanes 11, 12, and 14, as well as in positive control, there was the size of target band. The corresponding PCR products of lanes 11, 12, and 14 were gelatinized, cloned, and sequenced by TA. By comparing the results of sequencing, it was found that lane 12 was a rabbit antibody sequence and paired with light chain. This sequence was named GPC1-12. The next step was to construct GPC1-12 on the vector for expression.

### 3.4. ELISA Detection of Antibody Transfection Supernatant

**Figure 3:**
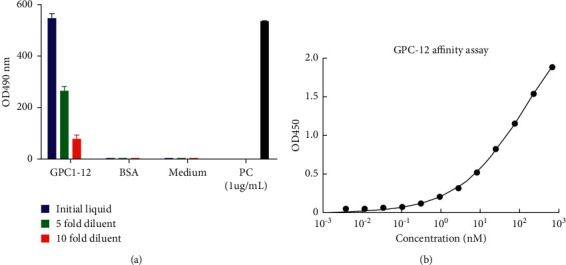
Detection of GPC1-12 in transfection supernatant was determined using ELISA and GPC1-12 affinity approaches. (a) Both BSA and medium were set as the negative control; PC (positive control): poly-IgG purified by affinity chromatography from positive rabbit serum. (b) GPC1-12 affinity determination. Enzyme-linked immunosorbent assay (ELISA) was performed to test the binding ability. The initial concentration of the coated antigen was 100 *μ*g/mL, and then it was double diluted. It was shown that compared with antigen and blank control groups, GPC1-12 antibodies purified can specifically bind to GPC1 antigens.

To understand the titer of antibody we produced, ELSIA was undertaken. As shown in Figure 3(a), bovine serum albumin (BSA) and cell culture medium (Medium) were negative controls, and positive control (abbreviated as PC) was polyclonal antibodies purified from rabbit serum by affinity purification column. The expression of GPC1-12 was detected in the supernatant of transfection. The next step was affinity purification of the supernatant.

### 3.5. Affinity Determination of the Purified Antibody

In order to further verify the binding activity of the purified antibody and GPC1 antigen, we carried out ELISA binding test. The initial concentration of the purified antibody was 100 *μ*g/mL, and then it was diluted twice. The results showed that, as shown in Figure 3(b), compared with the uncoated antigen group and blank control, GPC1-12 purified antibody was shown to have specific binding performance with GPC1 antigen.

### 3.6. Detection of Antibody Binding Abilities to Natural Surface Protein

**Figure 4:**
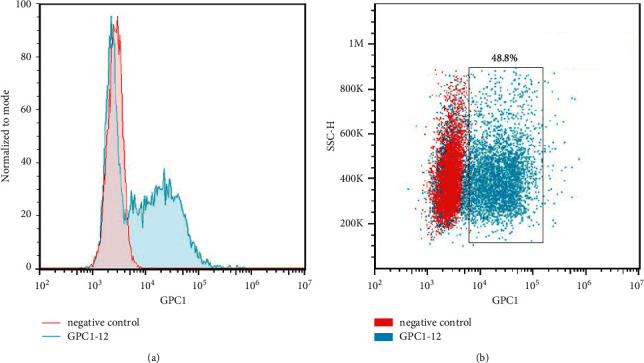
Feasibility of monoantibody GPC1-12 we obtained was validated using surface natural protein from the MDA-MB-231 cell line. The blue line represents monoantibody GPC1-12; the red line represents the negative control.

To verify the affinity of antibody we generated, evaluation was performed with flow cytometry. As shown in Figure 4, GPC1-12 monoclonal antibody (blue solid line) could bind well to cells but had a significant positive deviation from the negative control (red line).

## 4. Discussion

Pancreatic cancer is a highly malignant tumor, ranking near the eighth place among all kinds of lethal tumors. In addition, its cure rate and five-year survival rate are very low. Therefore, early diagnosis of pancreatic cancer is of particularly import, and there is a huge potential demand in patients with pancreatic cancer. Biomarker-based early diagnosis of cancer has always been prevailing, such as CA19-9-based diagnostic kit, but its specificity and sensitivity for the diagnosis of pancreatic cancer still need to be improved. It has been reported that GPC1 has a higher specificity for pancreatic cancer than CA19-9 [7]. The aim of this study was to develop and establish one effective method to screen out the more specific diagnostic antibodies for pancreatic cancer GPC1 by means of flow cytometry and single-cell amplification.

Similar to our previous report regarding single-cell study [16], three batches of rabbit single B cells were selected, 17 antibody sequences against human GPC1 were screened, and using these sequences, expression vectors were constructed, and antibodies were expressed. GPC1-12 was turned out to be the best antibody sequence in this screening process in our setting, which guaranteed the affinity of antibody antigen and the relative expression quantity. It was proved to be higher than those of other clones. There might be several reasons for the low expression of other antibodies. Firstly, after many rounds of PCR reaction, mutation could occur to some key bases of the antibody gene, which would greatly affect its expression level; secondly, the commercial expression vector pFUSE-rabbit Fc might have low expression efficiency, and other expression vectors could be replaced, such as pc-DNA serial vectors.

Traditional methods of antibody preparation mainly include hybridoma cell method and phage display method [17]. Outside of animal immunity, both of them have a lot of library building and screening work, which lasts for several months or even half a year. In this study, the period of antibody screening using immune antigens can be directly and remarkably shortened with the aid of flow cytometric cell sorting; moreover, the B cell clones isolated need not be cultured and expanded any more. The advantage of the methods we established here is that the amplification of antibody heavy chain and light chain genes can be directly obtained. It can directly obtain the matching information of antibody heavy chain and light chain, thus eliminating the cumbersome steps of cDNA library establishment and cell culture, therefore substantially greatly improving the efficiency of screening antibodies.

Based on the sequence of the antibody obtained in this experiment, the experiment that follows need to be considered, including the following: Firstly, the screened antibodies could be used to prepare early diagnostic kits for pancreatic cancer based on ELISA or flow cytometry. Secondly, with going deep in the research work, it has been reported that exosomes played an increasingly important role in the diagnosis and treatment of diseases, especially in cancer [18]. Considering this, the antibodies we screened can be used to isolate the antigen-specific exosomes by flow cytometry for subsequent clinical diagnosis and treatment. In conclusion, the method we established and developed here obtained highly specific antibody sequences and was able to substantially shorten the experimental cycle, which would be also suitable for screening other targets one is interested in.

## Figures and Tables

**Table 1:** Primer name and sequence.

Name	Sequence (5′ to 3′)
TSO^[12]^	AAGCAGTGGTATCAACGCAGAGTACrGrG + G^a^
Oligo(dT)18	TTTTTTTTTTTTTTTTTT
rIgH-Ld1	CTTCTCCTGGTCGCTGTGCT
rIgH-FR1	CACTCACCTGCACAGYCTCTGGA
rIgK-Ld1	CTGCTGGGGCTCCTGCT
rIgK-FR1	GCTGTGGGAGGCACAGTCAC
rIgL-Ld1-1	CCTCACAGTCCTGGCTCACTGCACA
rIgL-Ld1-2	CCYCCTCCTCKCTCACTGCACA
rIgH-J	TGARGAGAYRGTGACSAGGGT
rIgK-J-1	CGACGACCACCTYGGTCC
rIgK-J-2	TGATTTCCACCTTGGTGCC
rIgL-J	CTGTGACGGTCAGCTKGGTC
rIgHG-C1	GAAGACTGAYGGAGCCTTAGGT
rIgK-C1	GGTGGGAAGATGAGGACAGTAG
rIgL-C1	CCTCTGAGGAGGGCGGRAACA

**Table 2:** Detection of the serum-positive conversion titer.

Rabbit number	Detection of the antigen	Blank group	Negative group (1 : 1000)	Immune group (1 : 1000)	Immune group (1 : 4000)	Immune group (1 : 16000)	Immune group (1 : 32000)
Rabbit 1	GPC1	0.070	0.111	2.922	2.132	1.424	0.987
Rabbit 2	GPC1	0.062	0.082	2.536	2.071	1.054	0.663

## Data Availability

All data generated or analyzed during this study are truthfully presented. Relevant data are available upon request.

## Abbreviations

GPC1: Glypican-1
PBMCs: Peripheral blood mononuclear cells
ELISA: Enzyme-linked immunosorbent assay
FITC: Fluorescein-5-isothiocyanate
DMSO: Dimethyl sulfoxide
PBS: Phosphate-buffered saline
PCR: Polymerase chain reaction.
